# Improvement in Irretractable Pruritus With Intrahepatic Portosystemic Shunt Embolization: From MAID to a New Lease on Life

**DOI:** 10.1155/crhe/5822560

**Published:** 2026-02-18

**Authors:** Amitesh Bagha, Ali Helmi, Arash Jaberi, Gideon Hirschfield, Kristel Leung

**Affiliations:** ^1^ Division of Vascular and Interventional Radiology, University Health Network, University of Toronto, Toronto, Canada, utoronto.ca

## Abstract

Pruritus is a common, often debilitating symptom of liver disease. While most commonly seen in the setting of cholestasis and biliary obstruction, intrahepatic portosystemic shunts (PSSs) may also present with pruritus. The pathophysiology of intractable pruritus is not well understood and often requires multimodal management. We present a rare case of an 81‐year‐old woman with severe medically refractory pruritus with elevated serum bile acids in the absence of chronic liver disease. Antipruritics and plasmapheresis produced minimal relief, leading her to contemplate medical assistance in dying (MAID). Imaging during workup revealed two intrahepatic shunts, anomalously connecting the right portal and hepatic veins. Endovascular shunt embolization led to normalization of her serum bile acids and produced remarkable symptomatic relief. This highlights a rare but treatable cause of pruritus in an adult without chronic liver disease or biliary obstruction, while showcasing embolization as a safe and effective treatment strategy with significant improvement of the patient’s quality of life.

## 1. Introduction

Pruritus is a frequent, often debilitating symptom of liver disease secondary to cholestasis and biliary obstruction. Beyond the physical sequelae, chronic pruritus carries a profound psychiatric burden including depressed mood and even suicidal ideation [1]. The pathophysiology is not well elucidated and management remains challenging, often requiring a combination of topical therapeutics and oral agents, while advanced cases may require therapeutic plasma exchange or phototherapy. Shunt‐related pruritus is rarer and less well understood [2]. We present a rare case of severe medically refractory pruritus that remarkably resolved with embolization of two intrahepatic portosystemic shunts (PSSs) in an individual without established chronic liver disease.

## 2. Case Presentation

An 81‐year‐old female was referred to our institution for intractable pruritus that progressively worsened over 1.5 years. Her medical history was notable for moderate chronic kidney disease, hypertension, osteoporosis, and remote cholecystectomy. Initially seen at an outside institution for confusion, blood tests revealed elevated serum ammonia and mildly elevated total bilirubin levels (max 26 μmol/L), along with minimal elevated aminotransferases (max 48 U/L) for which she was started on lactulose. Given pruritus, bile acids were drawn and found to be persistently significantly elevated (70 to > 180 mIU/L). Workup for chronic liver diseases was unremarkable, including normal liver stiffness measurement, viral hepatitis, genetic metabolic conditions, and autoimmune liver disease; an extensive genetic cholestasis panel was also negative for any pathological alleles [3]. Imaging (CT and ultrasound) did not demonstrate any evidence of chronic liver disease, biliary obstruction, or portal hypertension.

**FIGURE 1:**
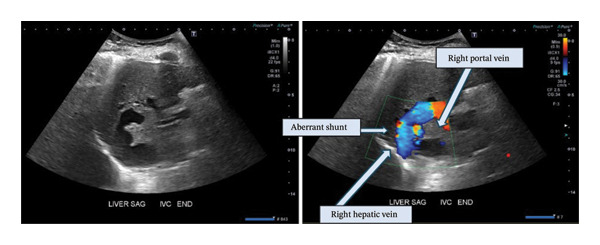
Representative ultrasound images of dominant right portal‐hepatic veno‐venous shunt in greyscale and color doppler.

The patient trialed several therapies for pruritus, including antihistamines, naltrexone, rifampicin, sertraline, and rifaximin, but did not continue these due to ineffectiveness or side effects (e.g., dizziness and fatigue). Moderate–severe itch burden and impact were routinely recorded with 5D itch scores ranging from 13 to 18 despite therapy. Cholestyramine and gabapentin provided only mild partial relief. Due to persistent debilitating symptoms and poor quality of life, the patient subsequently underwent plasmapheresis with minimal improvement. Given symptom severity and impact on the patient’s quality of life, she expressed interest in pursuing medical assistance in dying (MAID) (Figure 1).

MRI for biliary evaluation (to evaluate for sclerosing cholangitis) incidentally revealed an aberrant shunt between the right portal and right hepatic veins measuring 16 mm. The lesion was identified on retrospective review of CT colonography from 5 years ago, appearing slightly smaller previously. Doppler ultrasound revealed the following velocities: main portal vein: 33.2 cm/s, right portal vein: 39.7 cm/s, shunt: 45.2 cm/s, and right hepatic vein: 50.5 cm/s. Normal flow directions in the portal and hepatic veins were maintained with loss of the normal triphasic waveform in the right hepatic vein. There were no imaging findings to suggest choledocholithiasis, biliary sludge, intrahepatic biliary obstruction, or focal nodular hyperplasia.

Diagnostic venogram confirmed the presence of two adjacent shunts connecting the right portal and hepatic veins. A direct portal pressure of 13 mmHg, average free hepatic venous pressure of 8.3 mmHg, and average wedged hepatic vein pressure of 8.3 mmHg were measured. A portosystemic gradient of 4 mmHg was calculated using the difference of the right atrial and portal pressure.

**FIGURE 2:**
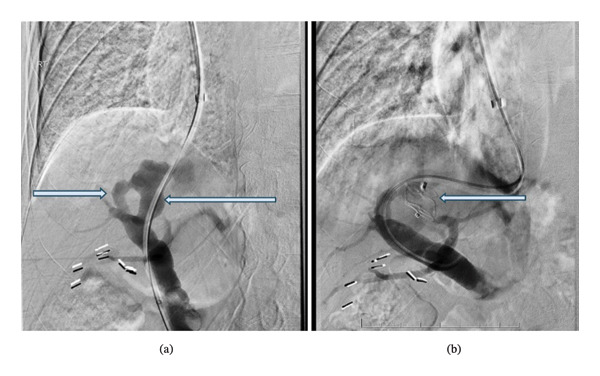
Representative pre‐embolization digital subtraction angiogram (a) demonstrating two aberrant vascular connections (arrows) between the right hepatic and right portal vein. Postembolization digital subtraction angiogram (b) demonstrating occlusion of the larger shunt with an Amplatzer Plug (arrow).

Via a right trans‐jugular approach, a long 7 French sheath was advanced into the right hepatic vein. A combination of different wires and catheters were used to cross the smaller shunt into the right portal vein before subsequent cannulation and crossing of the larger shunt back into the portal vein for embolization. Once retrograde through‐and‐through access was obtained, satisfactory embolization of the larger shunt was performed via deployment of a 16‐mm Amplatzer Vascular Plug II (AVP 2, St. Jude Medical, St. Paul, MN) (Figure 2)

As postembolization ultrasound was not performed, interval changes in portal and hepatic venous flow velocities and waveforms after shunt closure were not directly assessed. However, within 1 month of embolization, serum bile acids fell rapidly to 16.7 mIU/mL, along with resolution of pruritus (with objective decline in 5D itch score to 11). Six months postprocedure, bile acids remained below 20 mIU/mL with significant corresponding improvement in pruritus evidenced by discontinuation of cholestyramine and reduced gabapentin dosage. Total bilirubin and transaminase levels have remained normal > 1 y following shunt closure. Lactulose was discontinued without recurrent encephalopathy. At 1‐year follow‐up postprocedure, the patient notes only occasional pruritus “once every few months” with minimal impact on quality of life.

## 3. Discussion

Intrahepatic PSSs are anomalous vascular connections between an intrahepatic portal vein and a systemic hepatic vein. Intrahepatic PSS may be congenital or acquired and be asymptomatic or present with pain, bleeding, or sequelae of hepatic dysfunction including jaundice and encephalopathy [4]. In this case, no history of abdominal trauma, prior hepatic intervention, malignancy, cirrhosis, or portal hypertension was identified, and the shunt was anatomically remote from the site of prior cholecystectomy, making an acquired or iatrogenic cause unlikely. A congenital shunt may be possible, with significantly delayed symptomatic manifestations in life.

The pathophysiology of pruritus in liver disease is complex and poorly understood, even in the setting of biliary obstruction or chronic liver disease. Bile acids are recognized as a key contributor, and bile acid sequestrants are a first‐line treatment for cholestatic pruritus [4]. In the setting of PSS, bile acids—normally carried from the gastrointestinal tract via the portal vein to the liver as part of enterohepatic circulation—aberrantly bypass the liver and enter systemic circulation via the shunt, resulting in elevated serum bile acids. Although their role in noncholestatic shunt‐related pruritus remains poorly established, other potential pruritogenic mediators may also have been contributing, which include the autotaxin‐lypophosphatidic acid signaling axis and interleukin‐31‐mediated neuromodulation [1, 5–7]. This may explain the presence of pruritus in the absence of liver failure or cholestasis in our patient, as well as noted in prior reports of PSS [2, 8].

The normalization of serum bile acids and resolution of pruritus following shunt embolization strongly supports a causal relationship between the portosystemic shunting and symptomatology, while acknowledging the possibility of contributory pruritogenic pathways that remain incompletely defined. The mechanism is akin to hepatic encephalopathy developing secondary to shunting and hepatic undersequestration of ammonia and other constituents of portal circulation [9]. For congenital intrahepatic PSS causing systemic complications, expert consensus recommends endovascular embolization for management, with single‐stage endovascular closure of intrahepatic CPSS when systemic complications are present, regardless of age [10]. We hypothesize that shunt closure redirects bile acids to be filtered to the liver for processing, thereby reducing serum levels and improving pruritus.

There are minimal reports of PSS presenting with pruritus in adults [11]. Surgical closure of a tumor‐associated PSS in a child resulted in improved pruritus, as did surgical closure of a PSS in a 22‐week‐old dog with elevated bile acids [8, 12]. There are also case reports highlighting the successful management of other PSS‐associated symptoms such as encephalopathy with embolization [13, 14]. This is the first reported case demonstrating intrahepatic PSS associated with severe intractable pruritus in an older adult without established chronic liver disease or biliary obstruction, with subsequent significant improvement and resolution of symptoms after successful embolization of the PSS.

## Funding

No funding was received for this manuscript.

## Consent

All the patients allowed personal data processing and informed consent was obtained from all individual participants included in the study.

## Conflicts of Interest

The authors declare no conflicts of interest.

## Data Availability

Data sharing is not applicable to this article as no datasets were generated or analyzed during the current study.
